# Investigation of Azimuth Multichannel Reconstruction for Moving Targets in High Resolution Wide Swath SAR

**DOI:** 10.3390/s17061270

**Published:** 2017-06-02

**Authors:** Weixian Tan, Wei Xu, Pingping Huang, Zengshu Huang, Yaolong Qi, Kuoye Han

**Affiliations:** 1 College of Information Engineering, Inner Mongolia University of Technology, Hohhot 010051, China; hwangpp@imut.edu.cn; 2 Inner Mongolia Key Laboratory of Radar Technology and Application, Hohhot 010051, China; 3 Department of Spaceborne Microwave Remote Sensing, Institute of Electronics, Chinese Academy of Sciences (IECAS), Beijing 100190, China; xuwei2011@mail.ie.ac.cn; 4 Electronics & Information Engineering, Beihang University, Beijing 100191, China; zengshu_huang@163.com (Z.H.); longgniy@163.com (Y.Q.); 5 China Electronics Technology Group Corporation, Information Science Academy, Beijing 100098, China; kuoyehan@hotmail.com

**Keywords:** high resolution wide swath (HRWS), azimuth multichannel reconstruction, moving target imaging, target velocity estimation, synthetic aperture radar (SAR)

## Abstract

The azimuth multichannel imaging scheme with the large receive antenna divided into multiple sub-apertures usually leads to azimuth non-uniform sampling, and echoes from all azimuth channels should be reconstructed based on the signal model before conventional SAR imaging. Unfortunately, the multichannel signal model of a moving target is different from that of a fixed target. This paper analyzes the multichannel signal model of the moving target and the effect of the target velocity on azimuth multichannel reconstruction. Based on the multichannel signal mode of the moving target, a new multichannel signal reconstruction algorithm is proposed. Furthermore, the slant range velocity is estimated by computing signal energy distribution. Simulation results on point targets validate the proposed approach.

## 1. Introduction

Geometric resolution and swath width are two of most important parameters for spaceborne synthetic aperture radar (SAR) systems. However, they pose different pulse repetition frequency (PRF) requirements [1]. In the spaceborne SAR system, the wide swath requires the low PRF, while the high azimuth resolution needs the high PRF to ensure enough azimuth sampling. To implement the high resolution wide swath (HRWS) imaging capacity, this restriction could be overcome by dividing the large receive antenna in azimuth into multiple sub-apertures to receive echoes. The PRF could be low, but the azimuth effective sampling rate is increased according to the number of sub-apertures. To ensure the uniform azimuth sampling, the optimum PRF value should satisfy the following relationship:

(1) $${\mathrm{PRF}}_{opt}=\frac{2v_{s}}{N\cdot d}$$

where $v_{s}$ is the sensor velocity, N is the number of azimuth sub-apertures, and is the distance between two adjacent sub-apertures. However, in most cases, the optimum PRF in the HRWS SAR system couldn’t be selected due to the timing diagram selection. Therefore, echoes from all azimuth channels should be reconstructed to resolve the azimuth non-uniform sampling problem. Up to now, multiple azimuth multichannel reconstruction algorithms have been proposed for different SAR imaging modes based on the multichannel signal mode of the fixed target [2,3,4].

Compared with the multichannel signal model of the fixed target, the signal model of the moving target is obviously different. If the azimuth multichannel raw data of moving targets are still handled by the conventional azimuth multichannel reconstruction algorithms, the Doppler spectra of moving targets can’t be well recovered, which results in defocused moving targets and ghost targets. The defocusing problem for moving targets is mainly due to the changed relative velocity that cannot be well resolved by an auto-focusing approach similar to the single channel case [5,6,7,8,9]. Fortunately, there are many available autofocus algorithms for estimating the phase error caused by the slant range and the relative velocity, such as the phase gradient autofocus approach, the minimum entropy autofocus approach and other approaches [5,6,7,8,9]. Meanwhile, ghosts of the moving target are mainly caused by the phase mismatch in the azimuth multichannel SAR system due to the slant range velocity of the moving target [7]. Ghost targets of the moving target must be suppressed especially for moving ships imaging in ocean, even if their power are more than 20dB lower than their corresponding moving target.

In this paper, the azimuth multichannel signal model of the moving target is described, and imaging results of the azimuth multichannel raw data of moving targets handled by the conventional approach [3,4] for the fixed scene are presented. To reconstruct well azimuth signals of moving targets in the HRWS SAR, the Matched Reconstruction Filter Bank (MRFB) is proposed [10,11], and it introduces adapted reconstruction filters to obtain the signal with the highest signal to clutter plus noise ratio (SCNR) in the matched filter map. Therefore, the MRFB can be considered as a maximum-likelihood estimator. Different the MRFB method, a new azimuth multichannel reconstruction algorithm for moving targets based on the multichannel signal model of the moving target is proposed. The key point of the proposed approach is the multichannel phase calibration based on the signal mode of the moving target. Another important processing step of azimuth multichannel reconstruction for moving targets is the target velocity estimation. This paper proposes a new approach of the target slant range velocity estimation based on the energy distribution of the azimuth reconstructed signal in the Doppler domain. Afterwards, the resulting multichannel raw data for moving targets would be well reconstructed.

This paper is arranged as follows: in Section 2, the signal model of the moving target in the HRWS SAR system is described, while its corresponding properties and processing results by the conventional approach are analyzed. Section 3 is focused on presenting the proposed azimuth multichannel reconstruction approach. Simulation experiments on point targets are carried out in Section 4 to validate the proposed approach. Finally, this paper is concluded in Section 5.

## 2. Signal Model and Properties

In this section, the signal mode of a moving target in an azimuth multichannel SAR system for HRWS imaging is described. In the following, the focus is turned to the azimuth signal component of the raw data. Compared with the multichannel impulse response in azimuth of a fixed target, the multichannel phase mismatch phenomenon is analyzed.

### 2.1. Signal Mode

**Figure 1:**
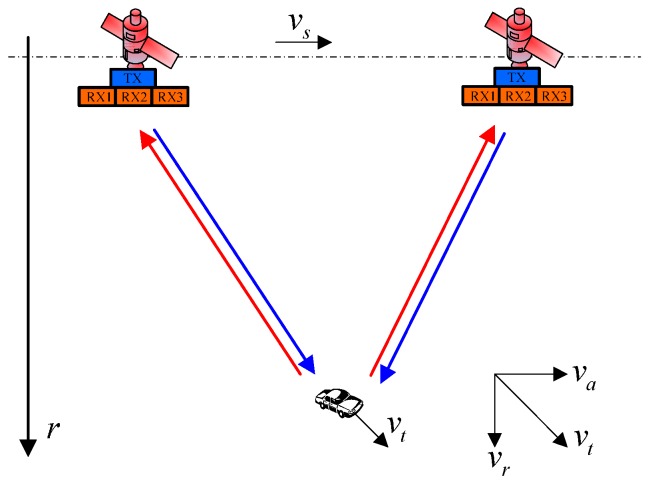
Imaging geometry of the slant-range plane for a moving target in an azimuth multichannel SAR system.

Figure 1 shows the imaging geometry of the slant-range plane for a moving target in an azimuth multichannel SAR system. During the SAR raw data collection for the azimuth multichannel SAR system, the target is with a constant velocity *v_t_* and the SAR platform velocity is *v_t_*. As shown in Figure 1, the slant-range velocity component and the along track velocity component are *v_r_* and *v_a_*, respectively.

For a given receive sub-aperture *n* separated by $\Delta x_{n}$ in azimuth from the transmit sub-aperture, the demodulated baseband signal from a single moving target can be expressed as:

(2) $$s_{n}(\tau ,t)=A\cdot w_{r}\left(\tau -\frac{R(t)+R_{n}(t)}{c}\right)\cdot w_{a,n}(t)\cdot \exp\left\{-j\frac{2\pi }{\lambda }\left(R(t)+R_{n}(t)\right)\right\}\cdot \exp\left\{jK_{r}{\left(\tau -\frac{R(t)+R_{n}(t)}{c}\right)}^{2}\right\}$$

with:

(3) $$R(t)=\sqrt{{\left(r+v_{r}t\right)}^{2}+{\left(v_{s}-v_{a}\right)}^{2}t^{2}}$$

(4) $$R_{n}(t)=\sqrt{{\left(r+v_{r}t\right)}^{2}+{\left(v_{s}t-v_{a}t-\Delta x_{n}\right)}^{2}}$$

where *A* is a complex constant, $w_{r}\left(\cdot \right)$ indicates the transmit pulse envelope, τ is the fast time, c is the light speed, $w_{a,n}(\cdot )$ indicates the azimuth antenna pattern, t is the slow time, λ is the wavelength, $K_{r}$ is the transmitted pulse chirp rate, r is the slant range, R(t) and $R_{n}(t)$ denote the range from the moving target to the transmitter and the receive sub-aperture *j*, respectively. The multichannel impulse response in azimuth is described as:

(5) $$h_{s,n}(t)=\exp\left\{-j\frac{2\pi }{\lambda }\left[\sqrt{{\left(r+v_{r}t\right)}^{2}+{\left(vt-\Delta x_{j}\right)}^{2}}+\sqrt{{\left(r+v_{r}t\right)}^{2}+{\left(vt\right)}^{2}}\right]\right\}$$

where $v=v_{s}-v_{a}$ is the relative along track velocity between the sensor and the moving target. Taking Taylor expression of both ranges R(t) and $R_{n}(t)$ from (5) to the order of two yields the quadratic approximation as:

(6) $$\begin{array}{ll} h_{s,n}(t) & \approx \exp\left\{-j\frac{4\pi }{\lambda }\left[r+\frac{(v^{2}+2v_{r}^{2})t^{2}-(v\Delta x_{n}-2v_{r}r)t+\Delta x_{n}^{2}}{r+v_{r}t}\right]\right\}\\ & =\exp\left(-j\frac{4\pi }{\lambda }r\right)\cdot \exp\left\{-j\pi \frac{2(v^{2}+2v_{r}^{2})}{\lambda (r+v_{r}t)}{\left[t-\frac{v\Delta x_{n}-2v_{r}r}{2(v^{2}+2v_{r}^{2})}\right]}^{2}\right\}\cdot \exp\left[-j\frac{2\pi \Delta x_{n}^{2}}{\lambda (r+v_{r}t)}\right]\cdot \exp\left[j\frac{2\pi }{\lambda }\frac{\left(v^{2}\Delta x_{n}^{2}-4vv_{r}r\Delta x_{n}+4v_{r}^{2}r^{2}\right)}{2(v^{2}+2v_{r}^{2})(r+v_{r}t)}\right]\; \end{array}$$

Due to *r* is much larger than $v_{r}t$ in spaceborne SAR, the approximated expression in (6) becomes:

(7) $$\begin{array}{ll} h_{s,n}(t) & \approx \exp\left(-j\frac{4\pi }{\lambda }r\right)\cdot \exp\left\{-j\pi \frac{2(v^{2}+2v_{r}^{2})}{\lambda r}{\left(t-\frac{v\Delta x_{n}-2v_{r}r}{2(v^{2}+2v_{r}^{2})}\right)}^{2}\right\}\\ & \cdot \exp\left(-j\frac{2\pi \Delta x_{n}^{2}}{\lambda r}\right)\cdot \exp\left(j\frac{2\pi }{\lambda }\frac{\left(v^{2}\Delta x_{n}^{2}-4vv_{r}r\Delta x_{n}+4v_{r}^{2}r^{2}\right)}{2(v^{2}+2v_{r}^{2})r}\right) \end{array}$$

Compared with the approximated multichannel impulse response of a fixed point target, the multichannel impulse response of a moving point target also evolves from the monostatic response but by a different relative velocity $v_{e}$, a different time delay $\Delta t_{n}$ and a different phase shift $\Delta \varphi_{n}$ as follows:

(8) $$v_{e}=\sqrt{v^{2}+2v_{r}^{2}}=\sqrt{{\left(v_{s}-v_{a}\right)}^{2}+2v_{r}^{2}}$$

(9) $$\Delta t_{n}=\frac{v\Delta x_{n}-2v_{r}r}{2(v^{2}+2v_{r}^{2})}\approx \frac{\Delta x_{n}}{2v}-\frac{v_{r}r}{v^{2}}$$

(10) $$\Delta \varphi_{n}=-\frac{2\pi \Delta x_{n}^{2}}{\lambda r}+\frac{2\pi }{\lambda }\frac{v^{2}\Delta x_{n}^{2}-4vv_{r}r\Delta x_{n}+4v_{r}^{2}r^{2}}{2(v^{2}+2v_{r}^{2})r}$$

As a result, the multichannel impulse response function in azimuth could be rewritten as follows:

(11) $$h_{s,n}(t)=h_{s}(t-\Delta t_{n})\cdot \exp(j\cdot \Delta \varphi_{n})$$

with:

(12) $$h_{s}(t)\approx \exp\left(-j\frac{4\pi }{\lambda }r\right)\cdot \exp\left\{-j\pi \frac{2\left(v^{2}+2v_{r}^{2}\right)}{\lambda r}t^{2}\right\}$$

### 2.2. Conventional Multichannel Reconstruction for Moving Targets

According to the above analyzed signal mode of the moving target in the HRWS SAR system, if we still adopt conventional azimuth multichannel reconstruction filters and imaging algorithms to handle the multichannel raw data for moving targets, the moving target would be defocused and ghost targets would occur. With the simulation parameters listed in Table 1, the effects of the along track velocity and the slant range velocity on moving target imaging are analyzed, respectively.

**Figure 2:**
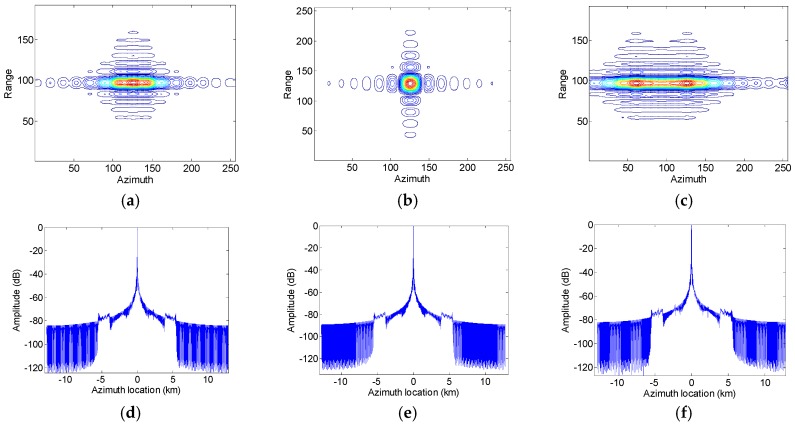
Imaging results of a point moving target with different along track velocities in the azimuth multichannel SAR system. (**a**–**c**) shows imaging results of moving targets with the along track velocity of −5 m/s, 0 m/s and 10m/s, respectively, while (**d**–**f**) shows the azimuth slice of the imaged target of (**a**–**c**).

Figure 2 shows imaging results of moving targets with different along-track velocities processed by the conventional approach for fixed targets. It can be seen that the along track velocity of the moving target leads to the imaged target being defocused. For the defocusing phenomenon of the imaged moving targets in the ocean, this problem could be easily resolved by the auto-focusing approach. Furthermore, the along-track velocity introduces the ghost targets due to the changed relative along-track velocity between the platform and the moving target. However, these ghost targets caused by the along-track velocity difference could be neglected as shown in Figure 2d–f.

**Figure 3:**
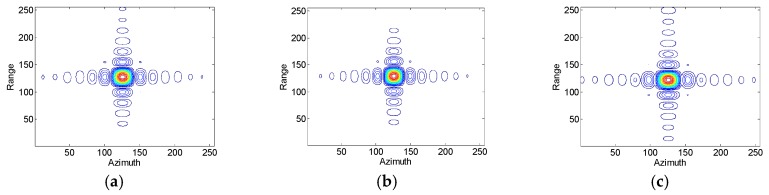

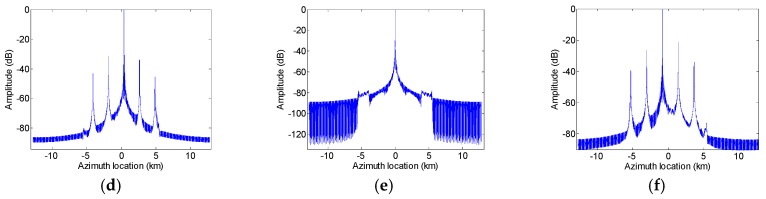
Imaging results of a point moving target with different slant range velocities in the azimuth multichannel SAR system. (**a**–**c**) shows imaging results of moving targets with the slant range velocity of −5 m/s, 0 m/s and 10 m/s, respectively, while (**d**–**f**) shows the azimuth slice of the imaged target of (**a**–**c**).

Figure 3 shows imaging results of moving targets with different slant-range velocities processed by the azimuth multichannel imaging approach for fixed targets. It can be seen that the slant-range velocity of the moving target leads to the imaged target being little defocused, an azimuth location shift as well as ghost targets as shown in Figure 3. Compared with imaging results shown in Figure 2 with the same velocity value, the effect of the slant range velocity of the moving target on target imaging quality parameters such as resolution, peak sidelobe ratio (PSLR) and integrated sidelobe ratio (ISLR) is less than the effect of the along track velocity. However, the higher ghost targets caused by the slant range velocity as shown in Figure 3d–f can’t be neglected and should be suppressed to improve the final obtained SAR image quality.

Assuming that the slant range velocity of the target is 15 m/s, with the simulation parameters listed in Table 1, the relative velocity difference caused by the slant range velocity is only 0.015 m/s and could be neglected. According to (9), the azimuth location shift Δx as shown in Figure 3 could be computed as follows:

(13) $$\Delta x=\Delta t\cdot v_{s}=-\frac{v_{s}v_{r}r}{{\left(v_{s}-v_{a}\right)}^{2}}$$

where Δt it the time shift caused by the target motion and is the second part of (9).

**Figure 4:**
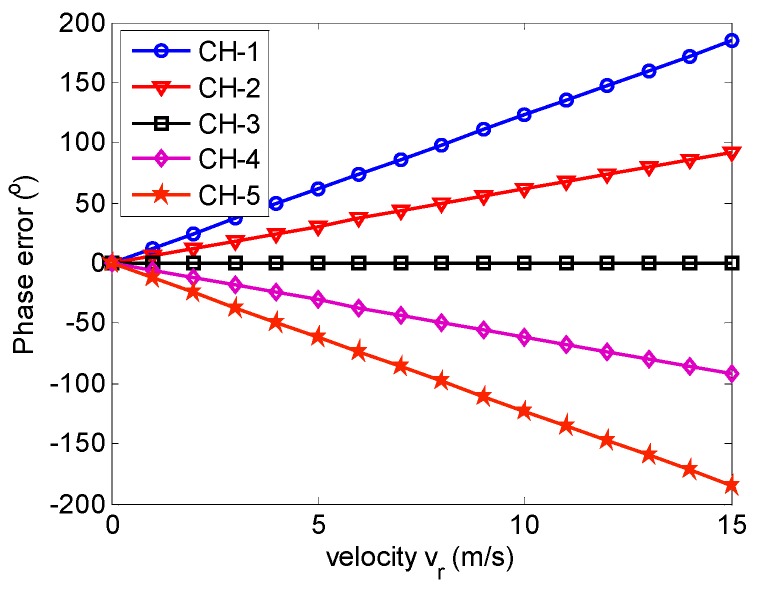
Multichannel phase mismatch caused by the slant-range velocity.

However, different phase shifts $\Delta \varphi_{n}$ in (9) in different azimuth receive channels lead to the receive channel imbalance for receiving echoes of moving targets, which results in multiple ghost targets in azimuth. With the simulation parameters listed in Table 1, Figure 4 shows the phase errors in different azimuth channels caused by the slant velocity of the moving target.

## 3. Azimuth Multichannel Imaging for Moving Targets

### 3.1. Azimuth Multichannel Reconstruction

Due to $v\gg v_{r}$, the approximated expression in (7) becomes:

(14) $$h_{s,n}(t)\approx \exp\left(-j\frac{4\pi }{\lambda }r\right)\cdot \exp\left\{-j\pi \frac{2v^{2}}{\lambda r}{\left(t-\frac{v\Delta x_{n}-2v_{r}r}{2v^{2}}\right)}^{2}\right\}\cdot \exp\left(-j\frac{\pi }{\lambda }\frac{v^{2}\Delta x_{n}^{2}+4vv_{r}r\Delta x_{n}-4v_{r}^{2}r^{2}}{v^{2}r}\right)$$

As a result, the multichannel response $H_{s,n}(f_{a})$ for a moving target could be also separated into the influence of the multichannel SAR system described by the function $H_{n}(f_{a})$ and the conventional monostatic SAR impulse response $H_{s}(f_{a})$ as follows:

(15) $$H_{s,n}(f_{a})\approx H_{n}(f_{a})\cdot H_{s}(f_{a})$$

with:

(16) $$H_{n}(f_{a})=\exp\left(-j\frac{\pi }{\lambda }\frac{v^{2}\Delta x_{n}^{2}+4vv_{r}r\Delta x_{n}-4v_{r}^{2}r^{2}}{v^{2}r}\right)\cdot \exp\left[-j2\pi f_{a}\frac{v\Delta x_{n}-2v_{r}r}{2v^{2}}\right]$$

where $H_{s}(f_{a})$ is the Fourier Transform of $h_{s}(t)$. Similar to the multichannel mode of the fixed target, a compact characterization of the whole multichannel system for the moving target is given by the system matrix $H(f_{a})$ as follows:

(17) $$H(f_{a})=\left[\begin{array}{ccc} H_{1}(f_{a}) & \cdots & H_{N}(f_{a})\\ H_{1}(f_{a}+\mathrm{PRF}) & \cdots & H_{N}(f_{a}+\mathrm{PRF})\\ \vdots & \ddots & \vdots \\ H_{1}(f_{a}+(N-1)\cdot \mathrm{PRF}) & \cdots & H_{N}(f_{a}+(N-1)\cdot \mathrm{PRF}) \end{array}\right]$$

Consequently, similar to the fixed scene case in the HRWS SAR [3], the azimuth multichannel reconstruction filters are also described by the matrix $P(f_{a})$ as follows:

(18) $$P(f_{a})=H^{-1}(f_{a})$$

Afterwards, the aliased Doppler spectra of all individual are combined together to recover the whole Doppler spectrum of the moving target.

### 3.2. Slant-Range Velocity Estimation

From (15), it can be seen that the along track velocity $v_{a}$ and the slant range velocity $v_{r}$ should be first known to compute the multichannel reconstruction matrix $P(f_{a})$. As mentioned, the along track velocity $v_{a}$ could be estimated by the auto-focusing approach similar to the single channel case and it makes much less contribution than the slant range velocity to the ghost targets. Therefore, only the slant range velocity estimation should be considered in this paper.

**Figure 5:**
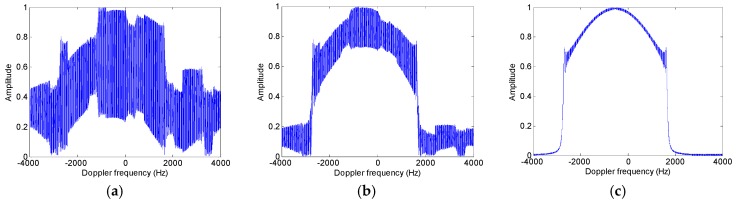

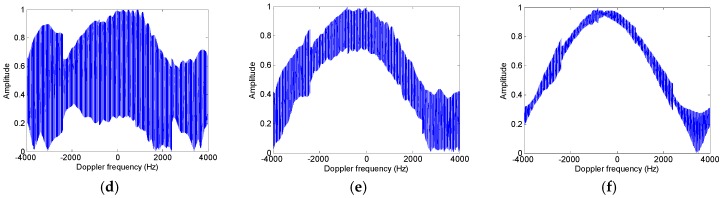
Echo energy distribution of a moving target in the Doppler domain after azimuth multichannel reconstruction with different estimated range velocities. (**a**) The selected velocity of 0 m/s in the band-limited case; (**b**) The selected velocity of 8 m/s in the band-limited case; (**c**) The selected velocity of 8.4 m/s in the band-limited case; (**d**) The selected velocity of 0 m/s in the full-band case; (**e**) The selected velocity of 8m/s in the full-band case; (**f**) The selected velocity of 8.4 m/s in the full-band case.

A new slant range velocity estimation approach based on the energy distribution of the reconstructed azimuth multichannel signal in the Doppler domain. For a fixed target in the azimuth multichannel case, all energy of the received echoes in each channel are distributed on a band of PRF around a center Doppler frequency, and most of energy would be distributed on a band of $B_{a}$ around the Doppler centroid $f_{dc}$ after azimuth multichannel reconstruction, where $B_{a}$ is the processed Doppler bandwidth. However, for a moving target, both the azimuth multichannel reconstruction matrix in (18) and the target Doppler centroid $f_{dc}$ are related to the target slant range velocity $v_{r}$. According to parameters listed in Table 1, Figure 5 shows the reconstructed multichannel Doppler spectra of a moving target handled by the reconstruction matrix with different target slant range velocities, where the target slant range velocities is 8.4m/s. As shown in Figure 5 with both the Doppler band-limited and the Doppler full-band cases, it can be seen that the Doppler spectrum is well reconstructed and most of energy would be distributed on a band of $B_{a}$ around the target Doppler centroid $f_{dc}$ only when the target slant range velocity $v_{r}$ is accurately obtained.

**Figure 6:**
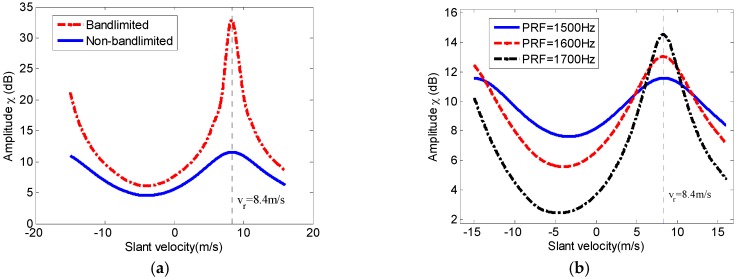
The relationship between the energy distribution factor χ with the estimated slant range velocity. (**a**) Band-limited and Non-band-limited cases; (**b**) Cases with different operated PRFs under the non-band-limited condition.

According to the phenomenon as shown in Figure 5, a new parameter named as energy distribution factor χ is introduced to describe the energy distribution of the reconstructed Doppler spectrum of the moving target as follows:

(19) $$\chi (v_{r})=\frac{\int_{-N\cdot \mathrm{PRF}/2+f_{dc}}^{N\cdot \mathrm{PRF}/2+f_{dc}}W(f_{a};v_{r})\cdot \mathrm{rect}\left[\frac{f_{a}-f_{dc}(v_{r})}{B_{a}}\right]\cdot df_{a}}{\int_{-N\cdot \mathrm{PRF}/2+f_{dc}}^{N\cdot \mathrm{PRF}/2+f_{dc}}W(f_{a};v_{r})\cdot df_{a}-\int_{-N\cdot \mathrm{PRF}/2+f_{dc}}^{N\cdot \mathrm{PRF}/2+f_{dc}}W(f_{a};v_{r})\cdot \mathrm{rect}\left[\frac{f_{a}-f_{dc}(v_{r})}{B_{a}}\right]\cdot df_{a}}$$

where $f_{dc}(v_{r})=2v_{r}/\lambda$ is the Doppler shift caused by the slant range velocity of the moving target, $W(f_{a};v_{r})$ is the reconstructed azimuth multichannel Doppler spectrum by the multichannel reconstructed matrix in (17) with the target slant range of $v_{r}$. Using the parameters listed in Table 1, Figure 6 shows the introduced energy distribution factor varying with the slant range velocity of the moving target in different cases. As a result, the slant range velocity of the moving target could be estimated by computing the factor $\chi (v_{r})$. Furthermore, the slant range velocity could be better estimated in the case of with the low Azimuth Ambiguity to Signal Ratio (AASR) level (band-limited or the large operated PRF) as shown in Figure 6, since the high AASR level would seriously affect the energy distribution in band of [−N⋅PRF/2,N⋅PRF/2].

## 4. Simulation Experiments

To validate the proposed multichannel processing approach for moving targets, simulation experiments on point targets are carried out in this section. Simulation parameters are listed in Table 1. Since the along track velocity of the moving target mainly leads to the imaged target being defocused and this problem is the same as the single channel case, we would just focused on the slant range velocity of the moving target.

**Figure 7:**
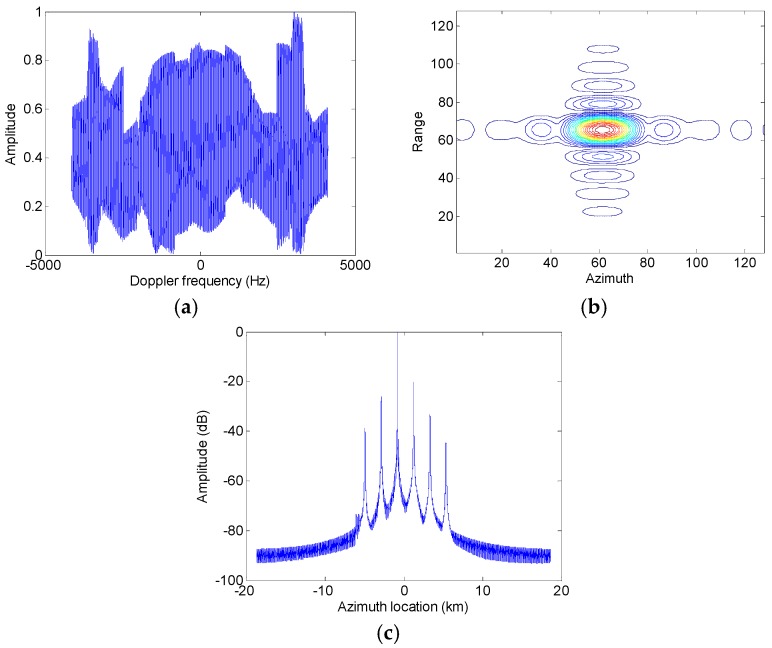
Imaging results of the moving target handled by the conventional approach. (**a**) The reconstructed Doppler spectrum; (**b**) The contour plots of the imaged moving target; (**c**) The azimuth slice of the imaged target.

**Figure 8:**
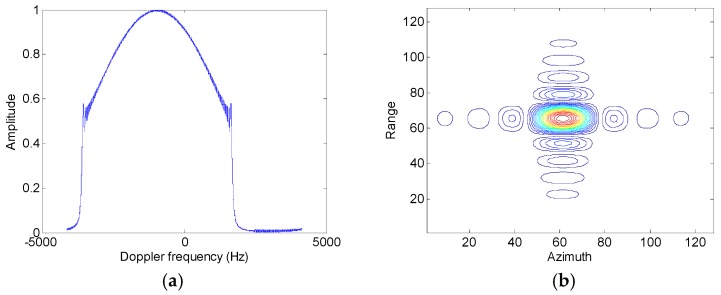

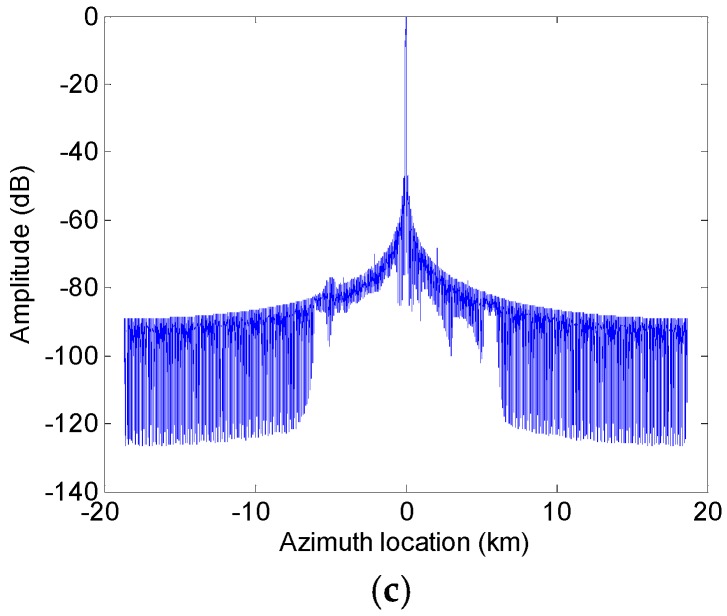
Imaging results of the moving target handled by the proposed approach. (**a**) The reconstructed Doppler spectrum; (**b**) The contour plots of the imaged moving target; (**c**) The azimuth slice of the imaged target.

**Figure 9:**
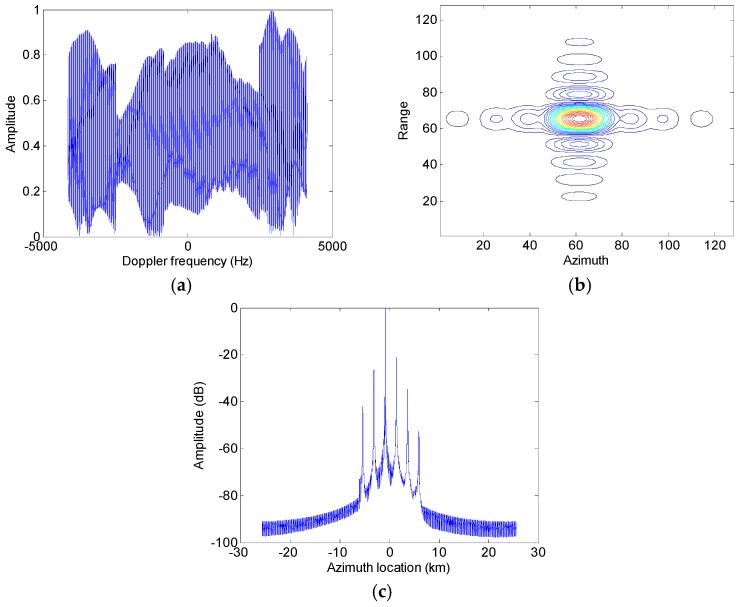
Imaging results of the moving target handled by the conventional approach. (**a**) The reconstructed Doppler spectrum; (**b**) The contour plots of the imaged moving target; (**c**) The azimuth slice of the imaged target.

**Figure 10:**
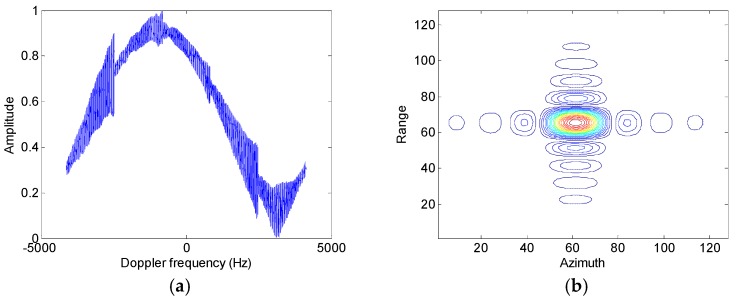

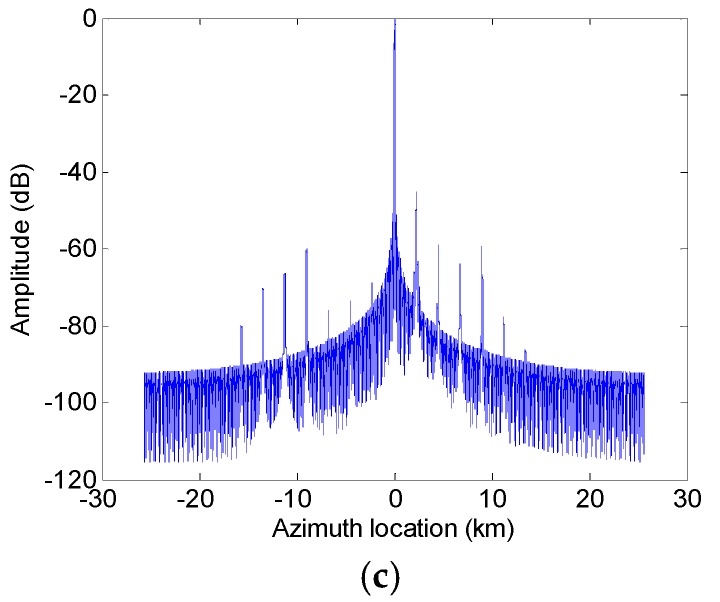
Imaging results of the moving target handled by the proposed approach. (**a**) The reconstructed Doppler spectrum; (**b**) The contour plots of the imaged moving target; (**c**) The azimuth slice of the imaged target.

Assumed that the slant range velocity is 10 m/s, Figure 7 and Figure 8 show simulation results of the moving target handled by the conventional azimuth multichannel reconstruction approach and the proposed approach, respectively. As shown in Figure 7 and Figure 8, the Doppler bandwidth is bandlimited. With the same parameters, Figure 9 and Figure 10 show results with the non-bandlimited Doppler spectrum.

**Figure 11:**
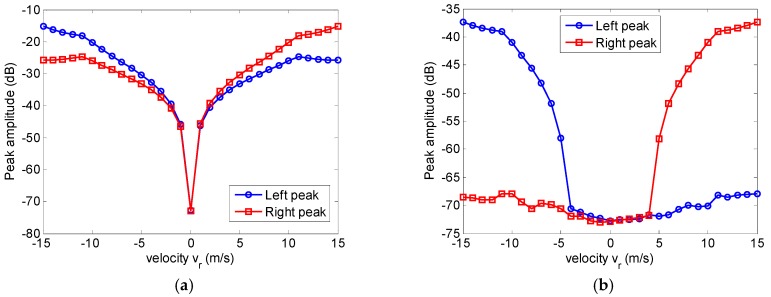
The maximum peak power of ghost targets in the moving target imaging. (**a**) The conventional approach; (**b**) The proposed approach.

Figure 11 shows the maximum peak power of ghost targets in the azimuth multichannel moving target imaging, and ghost targets are well suppressed (more than 20 dB) by the proposed approach as shown in Figure 11.

## 5. Conclusions

Azimuth multichannel with a large receive antenna divided into multiple sub-apertures will be adopted in future spaceborne SAR missions for the HRWS imaging capacity. However, the multichannel signal model of a moving target is quite different from that of a fixed target. If the conventional azimuth reconstruction algorithm is to handle the raw data of moving targets, multiple ghost targets would occur due to the azimuth multichannel imbalance caused by the slant range velocity of moving target. In this paper, an azimuth multichannel imaging approach for moving targets is proposed. The key points of the proposed approach are the moving target slant range velocity estimation and azimuth multichannel reconstruction based on the multichannel signal model of the moving target. Simulation results of point targets validate the proposed azimuth multichannel reconstruction approach for moving targets in high resolution wide swath SAR. Furthermore, for the case of moving targets on the ground, clutter suppression is required and moving targets are detected. For the case for ship monitoring at low sea states, clutter suppression is even not required. Afterwards, azimuth lines of interest lines for moving targets are picked out to avoid the degraded SAR image of stationary scatters, and then the selected raw data is handled by the proposed algorithm. Therefore, the proposed algorithm is carried out to handle the raw data of moving ships on sea.

## Figures and Tables

**Table 1:** System simulation parameters.

Simulation Parameter	Value
Carrier frequency	9.6 GHz
Equivalent transmit antenna length	3 m
The whole receive antenna length	10 m
Number of channels in azimuth	5
Transmitted pulse duration	4 μs
Transmitted pulse bandwidth	100 MHz
Sampling frequency	120 MHz
Operated system PRF	1600 Hz
Effective sensor velocity	7500 m/s
Slant range of swath center	600 km
